# Molecular identification in monophasic and nonmotile variants of *Salmonella enterica* serovar Typhimurium

**DOI:** 10.1002/mbo3.39

**Published:** 2012-11-26

**Authors:** M Bugarel, M-L Vignaud, F Moury, P Fach, A Brisabois

**Affiliations:** French Agency for Food, Environmental and Occupational Health and Health (ANSES), Laboratory for Food Safety23 Avenue du Général de Gaulle, 94706, Maisons-Alfort, France

**Keywords:** Genotyping, molecular markers, monophasic variants, nonmotile variant, serovar Typhimurium

## Abstract

Variant strains of *Salmonella enterica* serovar Typhimurium, lacking one or both flagellar phases have been widely reported. The monophasic *S*.1,4,[5],12:i:- variant has emerged worldwide in the past few years and has become one of the most frequently encountered in many countries. In contrast, monophasic *S*.1,4,[5],12:-:1,2 and nonmotile *S*.1,4,[5],12:-:- strains are rarely described. This study investigated seven molecular markers to identify and delineate monophasic *S*.1,4,[5],12:i:- (*n* = 90), *S*.1,4,[5],12:-:1,2 (*n* = 25), nonmotile *S*.1,4,[5],12:-:- (*n* = 17) strains, and some serovar Typhimurium strains (*n* = 124) collected through the French *Salmonella* network between 2001 and 2010. Three markers were commonly detected in serovar Typhimurium and in all variant strains: STM2757, *mdh* and *fliA-B*. Monophasic *S*.1,4,[5],12:i:- were genotypically confirmed by the absence of the *fljB*, *fljA*, and *hin* genes. Nevertheless, 13 (14.5%) of them were positive for these last three genes, revealing monophasic strains named “inconsistent” as previously described. All nonmotile 1,4,[5],12:-:- strains had the *fliC*, *fljA*, *fljB*, and *hin* genes and the *fliC* gene was detected in 88% of monophasic *S*.1,4,[5],12:-:1,2 strains. The combination of the seven markers detection enables to recognize eight different genotypes within the *S*.1,4,[5],12:i:- collection, among which the Spanish and the U.S. clones previously described could be distinguished and assigned to a genotype. Based on this molecular approach, 71% of the French *S*.1,4,[5],12:i:- collection belonged to the Spanish clone, whereas only 2% were assigned to the U.S. clone. This study highlights the usefulness of these molecular markers and genotypes for identifying lineages, especially among the epidemiologically important monophasic *S*.1,4,[5],12:i:- variant.

## Introduction

The main reservoir of *Salmonella enterica* is the intestinal tracts of various animal species and isolates are recovered from food and animal products such as raw meat, poultry products, and milk- and egg-based products (Swaminathan et al. 2006).

**Figure 1:**
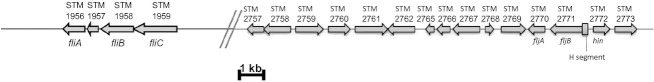
Map of the genomic regions carrying the targeted genes *fliA*, *fliB*, *fliC*, STM2757, *fljA*, *fljB*, and *hin* in the *Salmonella enterica* Typhimurium LT2 genome. Flagellin of the first flagellar phase is encoded by the *fliC* gene (STM1959). This gene constitutes an operon together with *fliA* (STM1956) and *fliB* (STM1958) genes. The expression of this *fliCBA* operon is under the negative regulation of FljA. The *fljA* (STM2770) gene forms an operon together with the *fljB* (STM2771) gene, encoding the flagellin of the second flagellar phase. The expression of this *fljBA* operon is regulated by the phase-inversion mechanism. The *fljBA* promoter is localized in the H segment, flanked by inverted sequences, *hixL* and *hixR*, between which site-specific recombination occurs carried out by the invertase Hin (STM2772).

This Gram-negative flagellated bacterium is responsible for salmonellosis, a food-borne disease causing enteric illness that can sometimes lead to hospitalization. In 2009, approximately 110,000 human cases of salmonellosis have been reported in Europe (EFSA and ECDC 2011). Within *S. enterica* species, more than 2600 serovars have been reported and the most frequently isolated in human cases worldwide are Enteritidis and Typhimurium. Together, they cause around 80% of confirmed cases of salmonellosis in Europe (EFSA and ECDC 2011). In France, 38.9% of human isolates collected by the National Reference Centre for *Salmonella* in 2009 (NRC-Salm) and 13% of food and in veterinary isolates collected by the French *Salmonella* Network were from serovar Typhimurium. *Salmonella* serovars are defined with an antigenic formula based on the presence of somatic and flagellar antigens according to the White–Kauffmann–Le Minor scheme (Grimont and Weill 2007). In most *S. enterica* subsp. *enterica* serovars, the antigenic formula is composed of two flagellar phases. The first flagellar phase is encoded by the *fliC* gene and the second one is encoded by the *fljB* gene. Expression of *fliC* and *fljB* is regulated through a mechanism called “phase variation” mediated by a DNA invertase, Hin, involved in the reversible inversion of the H segment, a DNA segment containing the promoter for the *fljB* gene (Fig. 1). This inversion occurs by site-specific recombination between two inverted repeat sequences flanking the H segment, *hixL* and *hixR* (Switt et al. 2009). The *fljA* gene, which encodes a negative regulator of *fliC* expression, is located downstream of *fljB*. When the H segment is in the “on” orientation, both *fljB* and *fljA* are transcribed. Only the second flagellar phase is expressed because *fliC* expression is repressed by FljA. However, when the H segment is in the “off” orientation, neither *fljB* or *fljA* are transcribed, so the *fliC* gene is expressed and only the first flagellar phase is phenotypically detected (Yamamoto and Kutsukake 2006). Moreover, a complete genomic sequence study revealed that the serovar Typhimurium strain LT2 harbored another DNA invertase gene named *fin* gene, and located to a 2.7-kb invertible DNA fragment within a resident prophage, Fels-2 (Kutsukake et al. 2006).

Monophasic variant lacking second flagellar phase *S*.1,4,[5],12:i:- emerged worldwide a few years ago (Switt et al. 2009) and have since become one of the most frequently isolated serovars in many countries. In France, this monophasic variant was the third most isolated serovar in humans in 2009, behind serovars Typhimurium and Enteritidis (F. X. Weill, S. Le Hello, Annual report from the NRC-Salm). In human cases as well as in food and animal products, isolation rates have increased 10-fold from 2005 to 2010 (NRC-Salm and ANSES *Salmonella* Network data). Furthermore, two other *S*.Typhimurium-like variants that are not frequently encountered have been described, one lacking the first flagellar phase *S*.1,4,[5],12:-:1,2 and the nonmotile variant, *S*.1,4,[5],12:-:-.

Numerous investigations on the emerging monophasic *S*.1,4,[5],12:i:- variant have been published (Echeita et al. 1999, 2001; Guerra et al. 2000; de la Torre et al. 2003; Mossong et al. 2007; Soyer et al. 2009; Bone et al. 2010; Hauser et al. 2010; Hopkins et al. 2010; Kozlica et al. 2010; Laorden et al. 2010; Trupschuch et al. 2010; Ido et al. 2011). In Europe, monophasic strains are often characterized by the antimicrobial resistance type to ampicillin, streptomycin, sulfonamides, and tetracycline (ASSuT) (Dionisi et al. 2009; Hauser et al. 2010) encoded by the *bla*_TEM_, *strA-strB*, *sul2,* and *tet*(B) genes, respectively (Hopkins et al. 2010; Lucarelli et al. 2010). These strains have been defined as the “European clone” (Hopkins et al. 2010) mainly having phage types DT193 and DT120 and carrying the newly described *Salmonella* genomic island 2 (Lucarelli et al. 2010). Two other lineages have also been described according to deletions in the genomic region between STM2691 and STM2774. The two previously described “U.S.” and “Spanish” clones are negative for *fljA* and *fljB* genes, whereas detection of *hin* gene is positive for the U.S. clone most often susceptible to antimicrobials and negative for the Spanish clone frequently associated to multiple antimicrobial resistance (Soyer et al. 2009). Interestingly, unexpected presence of *fljB* gene in some monophasic 1,4,[5],12:i:- strains has been described and these strains may represent an “inconsistent” variant of *S*.Typhimurium-like and have already been reported (Hopkins et al. 2010; Bugarel et al. 2012). Due to the increasing prevalence of monophasic *S*.1,4,[5],12:i:- isolates, particularly in the food chain, the European Food Safety Authority (EFSA) recently recommended the confirmation of the serological identification of monophasic *S*.1,4,[5],12:i:- strains using a polymerase chain reaction (PCR) protocol based on the detection of the *fliA-B* intergenic region and the *fljB* gene. Indeed, all serovar Typhimurium strains and its monophasic/nonmotile variants possess an IS200 fragment of 1 kb in the *fliA-B* intergenic region, which is not detected in the other serovars showing 250-bp products (EFSA 2010). Thus, this protocol aimed first to detect the phenotypically *S*.1,4,[5],12:i:- strains, which are not genotypically derived from serovar Typhimurium but from other serovars such as Lagos (1,4,[5],12:i:1,5), Agama (4,12:i:1,6), Farsta (4,12:i:e,n,x), Tsevie (1,4,12:i:e,n,z_15_), Gloucester (1,4,12,27:i:l,w), or Tumodi (1,4,12:i:z_6_), and second, to recognize the “true” *fljB*-positive *S*.1,4,[5],12:i:- strains from the “inconsistent” strains.

Therefore, the development of rapid methods adapted to the detection of such monophasic or nonmotile variants strains is critical for surveillance and identification of the different lineages is needed for food safety risk assessments and clinical microbiological investigations. In this study, a total of 256 strains of serovar Typhimurium, monophasic and nonmotile variant of *S*.Typhimurium-like isolated from food and animal origins were investigated. Seven molecular markers including those recommended by EFSA were used to improve the molecular characterization of the different variants and to determine the genetic profile of these strains.

## Materials and Method

### Bacterial strains and culture method

Two hundred and fifty-six *S. enterica* strains of serovar Typhimurium and its monophasic and nonmotile variants were investigated. These strains were isolated in France between 2001 and 2010 and collected through the *Salmonella* Network at the ANSES Laboratory for food safety, to which veterinary- and food-analysis laboratories regularly send *Salmonella* isolates. Isolates were selected so as to avoid duplicates and to cover the diversity of sources. They were recovered from various animal sources (*N* = 123): eagles (*n* = 1), cattle (*n* = 19), goats (*n* = 5), equines (*n* = 2), rabbits (*n* = 1), sheep (*n* = 6), parrots (*n* = 6), pigeons (*n* = 1), pigs (*n* = 11), snakes (*n* = 2), poultry (*n* = 67), slaughterhouse environment (*n* = 2); and from various categories of food products (*N* = 120): beef (*n* = 4), food-processing plants (*n* = 3), pork (*n* = 43), chicken (*n* = 28), cooked pork meat (*n* = 14), egg products (*n* = 1), ready meals (*n* = 7), seafood products (*n* = 1), dairy products (*n* = 18), and animal feed (*n* = 1). Thirteen isolates were collected from the natural environment: effluents (*n* = 7), rivers (*n* = 3), and mud (*n* = 3). Furthermore, the reference strain LT2 was used as a positive control for the investigated markers. Finally, a single isolate of each following serotypes Farsta, Gloucester, and Lagos was investigated.

### Conventional serotyping

The antigenic formulae of strains were serologically determined using agglutination tests with antisera (Bio-Rad Laboratories, Marnes-la-Coquette, France and AES, Bruz, France) as specified by the White–Kauffmann–Le Minor scheme (Grimont and Weill 2007) and according to a previously described in-house method accredited by the French Accreditation Committee (COFRAC, accreditation no. 1-2246, Section Laboratories, http://www.cofrac.fr) (Danan et al. 2009). The determinations of O and H antigens were performed using a pure culture grown on triple sugar iron (TSI) medium (AES, Bruz, France). Slide agglutination confirmed the absence of flagellar phase 1 or 2 after repeating agglutination assays at least three times using phase-inversion method. The collection included 125 isolates of serovar Typhimurium, 90 isolates of *S*.1,4,[5],12:i:-, 25 isolates of *S*.1,4,[5],12:-:1,2, 17 isolates of *S*.1,4,[5],12:-:-, and a single isolate of each following serovars: Farsta (4,12:i:e,n,x), Gloucester (1,4,12,27:i:l,w), and Lagos (1,4,[5],12:i:1,5). Different antigenic formulae were detected in monophasic and nonmotile isolates: 4,12:i:- (*n* = 69), 4,5,12:i:- (*n* = 21), 4,12:-:- (*n* = 7), 4,5,12:-:- (*n* = 6), 1,4,12:-:- (*n* = 4), 4,5,12:-:1,2 (*n* = 10), 1,4,12:-:1,2 (*n* = 10), and 4,12:-:1,2 (*n* = 5).

### PCR detection

Before DNA extraction, strains were cultured on Drigalski agar (BioMérieux, Marcy-l'Etoile, France). After overnight incubation at 37°C, DNA from a single pure colony was extracted using Instagene Matrix® (Bio-Rad Laboratories, Marnes-la-Coquette, France) according to the manufacturer's recommendations.

The *fliA-B* intergenic region and the *fliC* and *fljB* genes were detected with conventional PCR protocols based on EFSA recommendations (EFSA 2010) using the following primer pairs: FFLIB and RFLIA, Sense-59 and Antisense-83, and Sense-60 and Antisense-I, respectively. Each conventional PCR was performed in simplex using an Applied Biosystems Veriti cycler (Applied Biosystems, Carlsbad, California). PCR detection of the *fliA-B* intergenic region was performed in 1× PCR buffer, 3.5 mmol/L MgCl_2_, 0.3 mmol/L dNTPs, 0.6 μmol/L each primer (FFLIB and RFLIA) (EFSA 2010), and 1 U of FastStart Polymerase (Roche, Meylan, France). The cycling profile for this PCR involved denaturation at 94°C for 5 min, followed by 30 cycles of 94°C for 30 sec, 64°C for 30 sec, and 72°C for 90 sec, and a final step of 72°C for 10 min. PCR amplification of the *fljB* gene encoding a 1389-bp product linked to the phase-2 antigen determinant was performed in 1× PCR buffer, 2.5 mmol/L MgCl_2_, 0.3 mmol/L dNTPs, 1 μmol/L each primer (Sense-59 and Antisense-83) (EFSA 2010), and 1 U of FastStart Polymerase. The cycling profile for this PCR involved denaturation at 94°C for 5 min, followed by 30 cycles of 94°C for 30 sec, 62°C for 30 sec, and 72°C for 30 sec, and a final step of 72°C for 7 min. Amplification of the *fliC* gene encoding a 550-bp product linked to the phase-1 antigen determinant was performed in 1× PCR buffer, 1.5 mmol/L MgCl_2_, 0.2 mmol/L dNTPs, 0.5 μmol/L each primer (Sense-60 and Antisense-i) (Herrera-Leon et al. 2004; Levy et al. 2008), and 1 U of FastStart Polymerase. The cycling profile for this PCR involved denaturation at 94°C for 5 min, followed by 30 cycles of 94°C for 40 sec, 58°C for 20 sec, and 72°C for 20 sec, and a final step of 72°C for 7 min.

Moreover, real-time PCR reactions were designed to target the five following markers, the *ttrC* gene as *Salmonella* genus marker (Malorny et al. 2004), two serovar Typhimurium-associated genes: STM2757 and *mdh* (malic acid dehydrogenase gene) (Amavisit et al. 2005; Soyer et al. 2009; Hopkins et al. 2010), the *fljA* gene as *fliC* repressor and the *hin* gene as DNA invertase of the H segment (Soyer et al. 2009). PCR reactions were simultaneously performed using the Fluidgm Biomark™ real-time PCR system (Fluidigm®, San Francisco, California) with the 96.96 dynamic arrays™. This array system has the capacity to screen 96 DNA templates against 96 genetic markers in the same run. Amplifications were performed according to the manufacturer's recommendations using EvaGreen® DNA-binding dye (Biotium Inc, Hayward, California) and were followed by a melting curve analysis. The BioMark™ real-time PCR system was used with the thermal profile of 95°C for 10 min, followed by 35 cycles of 95°C for 15 sec, and 60°C for 1 min. Results were analyzed using the Fluidigm software packages “Fluidigm Melting Curve Analysis” (Version 3.0.2) and “Fluidigm Real-Time PCR Analysis” (Version 3.0.2).

### STM2757 sequencing

STM2757 was sequenced using STM2757-seqF (5′-TTC AGGT CGC TAC AGG CAA C-3′) and STM2757-seqR (5′-AGC AGC AAC TTG AAG TAT TGA CG-3′) primers. Locations of the STM2757-seqF and the STM2757-seqR primers are within the STM2756 (2894864–2894883) and close to the STM2758 in the intergenic region between STM2757 and STM2758 (2895789–2895768) in the *Salmonella* serovar Typhimurium LT2 sequence (AE006468), respectively. The cycling profile for this PCR involved denaturation at 98°C for 30 sec, followed by 35 cycles of 98°C for 10 sec, 60°C for 15 sec, and 72°C for 45 sec, and a final step of 72°C for 5 min.

### Sequences

Primer design and names were based on the nucleotide sequence of *S. enterica enterica* Typhimurium LT2 referenced in GenBank with the accession number AE006468. Primer sequences are listed in Table 1.

**Table 1:** Primer sequences designed for the seven markers amplification

Targets	Name	Primer sequence (5′…3′)	Location in AE006468	Size (bp)	Reference
Intergenic region of *fliA–fliB*	FFLIB	CTG GCG ACG ATC TGT CGA TG	2046411–2046392	964	EFSA (2010)
	RFLIA	GCG GTA TAC AGT GAA TTC AC	2045447–2045466		
*fljB*	Sense-59	CAA CAA CAA CCT GCA GCG TGT GCG	2914496–2914473	1389	EFSA (2010)
	Antisense-83	GCC ATA TTT CAG CCT CTC GCC CG	2913108–2913130		
*fliC*	Sense-60	ACT CAG GCT TCC CGT AAC GC	2048962–2048943	550	Herrera-Leon et al. (2004)
	Antisense-i	ATA GCC ATC TTT ACC AGT TCC	2048411–2048431		
*ttrC*	*ttrC*-F	CTC ACC AGG AGA TTA CAA CAT GG	1469436–1469414	94	Lucarelli et al. (2010)
	*ttrC*-R	AGC TCA GAC CAA AAG TGA CCA TC	1469342–1469364		
STM2757	STM2757-F	AAC CGT ACA GGG TTT ATA CGC C	2895201–2895222	91	This study
	STM2757-R	TTA TCG TGC CGC CGA ATT ATG G	2895292–2895271		
*mdh*	*mdh*-F	TGC CAA CGG AAG TTG AAG TG	3527121–3527102	260	Amavisit et al. (2005)
	*mdh*-R	CGC ATT CCA CCA CGC CCT TC	3526861–3526880		
*fljA*	*fljA*-F	TCC GAA GCC AGA ATC AAA TTT TCC	2912963–2912986	105	This study
	*fljA*-R	TAC GTT TTA ATG ATA TCC CTG TTC G	2913068–2913044		
*hin*	*hin*-F	CGC CCC GGC CTG AAA CGA	2914968–2914985	334	This study
	*hin*-R	CGA CTA ATC TGT TCC TGT TCA TGT T	2915302–2915278		

## Results

### Detection of markers common to serovar Typhimurium and to the variant strains

The 256 investigated strains as well as the LT2 strain, the Farsta, Gloucester, and Lagos were all positive for *ttrC,* which is a *Salmonella* genus marker. The three different markers (*fliA-B*, STM2757, and *mdh*) usually associated with serovar Typhimurium were explored on the same collection. As expected, the three strains of serovars Farsta, Gloucester, or Lagos were negative for these three markers (data not shown). The 124 strains of serovar Typhimurium all were positive for markers STM2757 and *mdh*. The 1-kb *fliA-B* intergenic region was also detected in all serovar Typhimurium strains except one. This latter *fliA-B*-negative strain was further serologically confirmed. All the *mdh-* and *fliA-B*-positive monophasic *S*.1,4,[5],12:i:- and *S*.1,4,[5],12:-:1,2 strains, were thus genotypically confirmed as variants of *S*.Typhimurium-like. Marker STM2757 only was detected in 92.2% (*n* = 83/90) and 92% (*n* = 23/25) of the *S*.1,4,[5],12:i:- and *S*.1,4,[5],12:-:1,2 strains, respectively. Six of the seven STM2757-negative *S*.4,12:i:- strains had been isolated from pork products, and the single remaining strain had been recovered from poultry. The two *S*.4,5,12:-:1,2 STM2757-negative strains had been isolated from effluents. The absence of detection of this marker was investigated using primers flanking STM2757 (STM2757-seqF and STM2757-seqR). The targeted locus cannot be amplified, revealing that the entire STM2757 ORF and its vicinity were absent (data not shown). Finally, all three Typhimurium-associated markers, *fliA-B*, STM2757, and *mdh*, were detected in the nonmotile strains (*n* = 17) (Table 2).

**Table 2:** Combination of seven markers-based patterns among serovar Typhimurium, monophasic and nonmotile strains

Antigenic formula	Number of isolates	Pattern name	STM2757	*mdh*	*fliA-B*	*fliC*	*fljB*	*fljA*	*hin*
1,4,[5],12:i:1,2	122 (98.4%)	P1	+	+	+	+	+	+	+
	1 (0.8%)	P2	+	+	−	+	+	+	+
	1 (0.8%)	P3	+	+	+	+	−	+	+
1,4,[5],12:i:-	13 (14.5%)	P1	+	+	+	+	+	+	+
	4 (4.4%)	P3	+	+	+	+	−	+	+
	4 (4.4%)	P4	+	+	+	+	+	−	−
	58 (64.5%)	P5	+	+	+	+	−	−	−
	3 (3.3%)	P6	+	+	+	+	−	+	−
	1 (1.1%)	P7	+	+	+	+	−	−	+
	6 (6.7%)	P8	−	+	+	+	−	−	−
	1 (1.1%)	P9	−	+	+	+	−	−	+
1,4,[5],12:-:1,2	22 (88%)	P1	+	+	+	+	+	+	+
	2 (8%)	P10	−	+	+	−	+	+	+
	1 (4%)	P11	+	+	+	−	+	+	+
1,4,[5],12:-:-	17 (100%)	P1	+	+	+	+	+	+	+

### Detection of the genes involved in flagellar structure (*fliC* and *fljB*) and in their expression regulation (*fljA* and *hin*)

All serovar Typhimurium strains including the reference LT2 strain, but one, carried both *fliC* and *fljB* genes encoding the first and second flagellar phases. Indeed, only a single Typhimurium strain did not display the *fljB* marker. This *fljB*-negative Typhimurium strain had been isolated from a ready-to-eat meal and was positive for the presence of the *fljA* and *hin* markers, targeting genes flanking *fljB* (Fig. 1). This strain was serologically confirmed as serovar Typhimurium, indicating that the second flagellar phase antigen was detected at the bacterial surface.

As expected, all *S*.1,4,[5],12:i:- strains carried the *fliC* gene involved in the structure of the first flagellar phase, and 81% (*n* = 73) of them were PCR negative for the gene encoding the second flagellar phase, *fljB* (Table 2). Thus, 17 phenotypically *S*.1,4,[5],12:i:- strains (18.9%) displayed the *fljB* marker, involving the presence of at least a part of the *fljB* gene.

The 25 *S*.1,4,[5],12:-:1,2 strains carried the gene involved in the structure of the second flagellar phase, *fljB* and 22 of them were *fliC* positive (Table 2). All the three remaining *fliC*-negative strains had been recovered from effluents.

All the 17 nonmotile *S*.1,4,[5],12:-:- strains carried both the *fliC* and *fljB* markers. These nonmotile strains also harbored a Typhimurium-like profile for the other flagellar antigenic markers. Of the 132 monophasic and nonmotile variant strains, 56 (43%) possessed the genetic content for the structure of both flagellar phases; 18.9% (17/90) of the *S*.1,4,[5],12:i:- strains, 88% (22/25) of the *S*.1,4,[5],12:-:1,2 strains, and 100% (17/17) of the *S*.1,4,[5],12:-:- strains.

The *fljA* and *hin* markers were perfectly conserved in all strains of serovar Typhimurium, *S*.1,4,[5],12:-:1,2 and *S*.1,4,[5],12:-:- (Table 2). In contrast, *fljA* and *hin* markers were generally not detected in *S*.1,4,[5],12:i:- strains. These results are fully coherent because *fljA* is located in the operon of the second flagellar phase and encodes the repressor of the expression of the first flagellar phase. These results are in total agreement with other previous studies (Echeita et al. 2001; Tavechio et al. 2004; Soyer et al. 2009; Hauser et al. 2010; Laorden et al. 2010; Trupschuch et al. 2010; Ido et al. 2011).

Furthermore, strains of serovars Farsta, Gloucester, and Lagos showed positive amplification of the *fliC* and *fljB* markers, which corroborates their phenotypic biphasic structure. The two markers *fljA* and *hin* were also detected in these last serovars (data not shown).

### Identification of marker-based patterns

The combination of presence and absence of the seven tested markers gave eleven patterns, P1–P11, for the set of 256 investigated strains listed in Table 2. Of the 124 strains of serovar Typhimurium, 122 showed pattern P1 (STM2757+ , *mdh*+, *fliAB*+, *fliC*+, *fljB*+, *fljA*+, and *hin*+); this pattern could be considered highly associated to serovar Typhimurium. Only two Typhimurium strains did not exhibit the P1 pattern; a single one lacking the *fliA-B* intergenic region of the first flagellar phase operon was assigned to P2 pattern, and the other *fljB*-negative strain was assigned to the P3 pattern (Table 2).

All the *S*.1,4,[5],12:-:- strains and the majority (88%) of *S*.1,4,[5],12:-:1,2 strains also displayed the P1 pattern, meaning that these strains are genotypically close to serovar Typhimurium. Of the three remaining *fliC*-negative *S*.4,5,12:-:1,2 strains, two of them were also STM2757 negative, displaying P10 pattern (STM2757−, *mdh*+, *fliAB*+, *fliC*−, *fljB*+, *fljA*+, and *hin*+)(Table 2).

In contrast, the *S*.1,4,[5],12:i:- strains investigated in this study exhibited eight different patterns. The most frequent P5 pattern (STM2757+, *mdh*+, *fliAB*+, *fliC*+, *fljB*−, *fljA*−, and *hin*−) was found in 58 (64.5%) strains. Such a pattern including the absence of *fljB*, *fljA*, and *hin* genes has been previously described and called “Spanish clone” by Soyer et al. (2009). Six other strains from food products, displaying the same pattern with the additional absence of STM2757 were assigned to P8 pattern (STM2757−, *mdh*+, *fliAB*+, *fliC*+, *fljB*−, *fljA*−, and *hin*−). These strains should also probably be related to the Spanish clone. Soyer et al. (2009) described deletions involving *fljA*, *fljB* genes, but leaving *hin* gene intact in *S*.1,4,[5],12:i:- strains. These strains were assigned to the U.S. clone. In our study, two such *fljA*- and *fljB*-negative strains were *hin* positive, suggesting they probably could be related to this U.S. clone. The STM2757 marker was detected in one of these two strains recovered from pork meat. This single strain were assigned to the P7 pattern (STM2757+, *mdh*+, *fliAB*+, *fliC*+, *fljB*−, *fljA*−, and *hin*+), whereas the other STM2757-negative strain was assigned to the P9 pattern (STM2757−, *mdh*+, *fliAB*+, *fliC*+, *fljB*−, *fljA*−, and *hin*+) (Table 2).

Moreover, this study identified three other patterns that have never been described before. The P4 pattern (STM2757+, *mdh*+, *fliAB*+, *fliC*+, *fljB*+, *fljA*−, and *hin*−), highly similar to those described in Spanish clone strains was observed in four strains from pork (*n* = 2) and chicken (*n* = 2) sources. The only difference lies in the presence of the *fljB* marker. Strains carrying this pattern should be subjected to numerous recombination events leading to the conservation of the *fljB* gene and deletion of the *fljA* and *hin* genes that flank it (Fig. 1). However, the absence of the *fliC* repressor gene (*fljA)* allows constitutive expression of the first flagellar phase. The absence of the *hin* invertase gene does not allow the switch between the expression of the first and second flagellar genes. Therefore, with regard to the serological result, the H segment may be locked in the “off” orientation (Fig. 1) (Yamamoto and Kutsukake 2006; Switt et al. 2009). Four other strains from pork products (*n* = 3) and poultry (*n* = 1) were assigned to P3 pattern (STM2757+, *mdh*+, *fliAB*+, *fliC*+, *fljB*−, *fljA*+, and *hin*+) related to the Typhimurium P1 pattern, differing only by the absence of the *fljB* gene. Finally, three monophasic *S*.1,4,[5],12:i:- strains recovered from pigs (*n* = 1), poultry (*n* = 1), and pork products (*n* = 1) exhibited the P6 pattern (STM2757+, *mdh*+, *fliAB*+, *fliC*+, *fljB*−, *fljA*+, and *hin*−).

## Discussion

This study aimed to investigate three different molecular markers already described for the identification of serovar Typhimurium.

One of these markers, the *fliA-B* intergenic region proposed by the EFSA working group (EFSA 2010) enables the identification of all serovar Typhimurium strains except one. This latter *fliA-B*-negative strain was further serologically confirmed to be serovar Typhimurium. The *fliA* gene encodes the flagellum-specific sigma factor σ^28^ of *Salmonella* Typhimurium (Ikebe et al. 1999) and *fliB* gene encodes a flagellin methylase (Frye et al. 2006). So, FliA regulates the expression of genes involved in the structure and the assembly of both flagella, whereas the flagellin methylation carried out by FliB is not involved in mobility. This methylation should be required for *Salmonella* virulence and not for flagellin function. The only marker detected in all serovar Typhimurium strains including the monophasic and nonmotile variants was *mdh*, whereas the STM2757 and *fliA-B* markers amplified in most but not all of these strains at a rate of 96.5% and 99.6%, respectively. Furthermore, after the investigation of the *mdh* marker on a collection of 937 strains belonging to a large panel of more than 230 different serovars, no cross-reaction was detected except for a single isolate of serovars Kibusi and New Mexico; both these serovars are very rarely reported worldwide.

Then, the implicated genes in the flagellar structure and the expression were investigated. While Typhimurium strains should possess all tested flagellar genes, a *fljB*-negative Typhimurium strain was identified. This strain was positive for the presence of the *fljA* and *hin* markers, targeting genes flanking *fljB* (Fig. 1) and has been serologically confirmed as serovar Typhimurium, indicating that the second flagellar-phase antigen was well detected at the bacterial surface. On the other hand, some monophasic *S*.1,4,[5],12:i:- strains were *fljB* positive. Such strains already reported by Hopkins et al., Soyer et al. and Bugarel et al. are considered to be “inconsistent” variants (Soyer et al. 2009; Hopkins et al. 2010; Bugarel et al. 2012) as they are characterized by an inconsistent serological detection of the second flagellar phase. These findings could suggest that such strains may have deletions or mutation in the *fljB* gene out of the primer-binding site of the *fljB* marker that modifies protein structure inhibiting serological detection. Nevertheless, sequencing data is requested to consolidate this hypothesis. Moreover, the invertible promoter controlling *fljB* and *fljA* expression should be locked in one position, allowing only the expression of the first flagellar phase as suggested by Hopkins et al. (2010). Another invertase, Fin, has been described in *Salmonella* Typhimurium LT2 reference strain. The *fin* invertase gene is located in the Fels-2 prophage at 34.5 kb of the *hin-fljBA* locus. Hin and Fin invertases share 62% of their amino acids and their hix and fix recombination sites share 81% of their nucleotide sequence (Kutsukake et al. 2006). In the absence of the Hin invertase gene, Fin can carry out the inversion of the H segment (Kutsukake et al. 2006).

In the same way, the divergence between the phenotypic and genotypic characterization of *S*.1,4,[5],12:-:1,2 strains could be explained by a deletion or a mutation in the *fliC* gene that does not affect the PCR amplification of the *fliC* marker, but affects serological detection of the surface antigen. Another explanation could be that the invertible promoter is locked in the gene activation position and thereby promotes *fljB* gene expression. A dysfunction of the promoter invertase Hin or a mutation in the *hix* recombination sites may be involved.

The phenotypically nonmotile strains also harbored a Typhimurium-like profile for the flagellar antigenic markers. Even if the four markers of the structure and expression regulation of both flagellar phases were detected, other explanations of such nonmotile function could be linked to the failure of some hook-basal body genes involved in the flagellar assembly.

Finally, combination of the seven molecular markers enables to distinguish 11 patterns, of which some of them could be related to some previously described lineages within the emerging monophasic *S*.1,4,[5],12:i:- variant. Thus, this study highlighted that 71% of the French *S*.1,4,[5],12:i:- strains assigned to P5 or P8 patterns could derive from the previously described Spanish clone, whereas only 2% of these monophasic strains assigned to P7 and P9 patterns could be attributed to the U.S. clone. Moreover, 13 *fljB*-positive *S*.1,4,[5],12:i:- strains (14.5%) carried the Typhimurium-associated P1 pattern (Table 2) gathering the “inconsistent” strains according to the Hopkins et al. definition (Hopkins et al. 2010). This particular group of strains was also found in one of our previous studies (Bugarel et al. 2012). Our investigation revealed for the first time the presence of the *hin* gene in the “inconsistent” *S*.1,4,[5],12:i:- group of strains. Thus, based on their assignment to pattern P1, such strains could be genotypically considered as Typhimurium-like strains with a dysfunction in gene expression. Therefore, absence of the phase-2 protein is not due to the absence of the encoding gene, but should be related to dysfunction in gene expression, in protein translocation, in flagellar filament assembly (Bugarel et al. 2012).

## Conclusion

Besides the identification of two previously described clones (U.S. and Spanish clones) within the French monophasic *S*.1,4,[5],12:i:- variant collection over the past 10 years, we evidenced three new, previously undescribed genotypes among the monophasic variant strains assigned to P3, P4, and P6 patterns. Inconsistent variants of serovar Typhimurium, serologically monophasic but genetically Typhimurium like, were observed in 14.5% (*n* = 13) of the French monophasic lacking the second flagellar phase. Furthermore, *S*.1,4,[5],12:-:1,2 and *S*.1,4,[5],12:-:- mainly carry the same P1 pattern as Typhimurium, in 88% and 100% of the investigated isolates, respectively, suggesting that these serologically monophasic or nonmobile strains are genetically Typhimurium-like. To test this hypothesis, *fliC*, *fljA*, *fljB*, and *hin* genes detected in the monophasic and nonmotile phenotypes variant strains need to be sequenced. Proteomic studies also could provide clues as to the functionality of the proteins involved. Finally, this study highlights the usefulness of the seven described molecular markers to delineate genotypes and identify lineages, especially among the epidemiologically important monophasic *S*.1,4,[5],12:i:- variant.

## References

[b1] Amavisit P, Boonyawiwat W, Bangtrakulnont A (2005). Characterization of *Salmonella enterica* serovar Typhimurium and monophasic *Salmonella* serovar 1,4,[5],12:i:- isolates in Thailand. J. Clin. Microbiol.

[b2] Bone A, Noel H, Le Hello S, Pihier N, Danan C, Raguenaud ME (2010). Nationwide outbreak of *Salmonella enterica* serotype 4,12:i:- infections in France, linked to dried pork sausage, March-May 2010. Euro. Surveill.

[b3] Bugarel M, Granier SA, Bonin E, Vignaud ML, Roussel S, Fach P (2012). Genetic diversity in monophasic (1,4,[5],12:i:- and 1,4,[5],12:-:1,2) and in non-motile (1,4,[5],12:-:-) variants of *Salmonella enterica* Typhimurium. Food Res. Int.

[b4] Danan C, Fremy S, Moury F, Bonhert ML, Brisabois A (2009). http://www.afssa.fr/cahierdelareference/numero2/PN6010htm.

[b5] Dionisi AM, Graziani C, Lucarelli C, Filetici E, Villa L, Owczarek S (2009). Molecular characterization of multidrug-resistant strains of *Salmonella enterica* serotype Typhimurium and monophasic variant (S. 4,[5],12:i:-) isolated from human infections in Italy. Foodborne Pathog. Dis.

[b6] Echeita MA, Aladuena A, Cruchaga S, Usera MA (1999). Emergence and spread of an atypical *Salmonella enterica* subsp. *enterica* serotype 4,5,12:i:- strain in Spain. J. Clin. Microbiol.

[b7] Echeita MA, Herrera S, Usera MA (2001). Atypical, *fljB*-negative *Salmonella enterica* subsp. *enterica* strain of serovar 4,5,12:i:- appears to be a monophasic variant of serovar Typhimurium. J. Clin. Microbiol.

[b8] EFSA (2010). Scientific opinion on monitoring and assessment of the public health risk of “*Salmonella* Typhimurium-like” strains. EFSA J.

[b9] EFSA and ECDC (2011). The European Union summary report on trends and sources of zoonoses, zoonotic agents and food-borne outbreaks in 2009. EFSA J.

[b10] Frye J, Karlinsey JE, Felise HR, Marzolf B, Dowidar N, McClelland M (2006). Identification of new flagellar genes of *Salmonella enterica* serovar Typhimurium. J. Bacteriol.

[b11] Grimont PAD, Weill FX (2007). Antigenic formulae of the Salmonella serovars. http://wwwpasteur.fr.ip/portal/action/WebdriveActionEvent/oid/01s-000036-089.

[b12] Guerra B, Laconcha I, Soto SM, Gonzalez-Hevia MA, Mendoza MC (2000). Molecular characterisation of emergent multiresistant *Salmonella enterica* serotype [4,5,12:i:-] organisms causing human salmonellosis. FEMS Microbiol. Lett.

[b13] Hauser E, Tietze E, Helmuth R, Junker E, Blank K, Prager R (2010). Pork contaminated with *Salmonella enterica* serovar 4,[5],12:i:-, an emerging health risk for humans. Appl. Environ. Microbiol.

[b14] Herrera-Leon S, McQuiston JR, Usera MA, Fields PI, Garaizar J, Echeita MA (2004). Multiplex PCR for distinguishing the most common phase-1 flagellar antigens of *Salmonella* spp. J. Clin. Microbiol.

[b15] Hopkins KL, Kirchner M, Guerra B, Granier SA, Lucarelli C, Porrero MC (2010). Multiresistant *Salmonella enterica* serovar 4,[5],12:i:- in Europe: a new pandemic strain?. Euro. Surveill.

[b16] Ido N, Kudo T, Sasaki K, Motokawa M, Iwabuchi K, Matsudate H (2011). Molecular and phenotypic characteristics of *Salmonella enterica* serovar 4,5,12:i: isolated from cattle and humans in Iwate prefecture, Japan. J. Vet. Med. Sci.

[b17] Ikebe T, Iyoda S, Kutsukake K (1999). Structure and expression of the *fliA* operon of *Salmonella typhimurium*. Microbiology.

[b18] Kozlica J, Claudet AL, Solomon D, Dunn JR, Carpenter LR (2010). Waterborne outbreak of *Salmonella* I 4,[5],12:i. Foodborne Pathog. Dis.

[b19] Kutsukake K, Nakashima H, Tominaga A, Abo T (2006). Two DNA invertases contribute to flagellar phase variation in *Salmonella enterica* serovar Typhimurium strain LT2. J. Bacteriol.

[b20] Laorden L, Herrera-Leon S, Martinez I, Sanchez A, Kromidas L, Bikandi J (2010). Genetic evolution of the Spanish multidrug-resistant *Salmonella enterica* 4,5,12:i:- monophasic variant. J. Clin. Microbiol.

[b21] Levy H, Diallo S, Tennant SM, Livio S, Sow SO, Tapia M (2008). PCR method to identify *Salmonella enterica* serovars Typhi, Paratyphi A, and Paratyphi B among *Salmonella* isolates from the blood of patients with clinical enteric fever. J. Clin. Microbiol.

[b22] Lucarelli C, Dionisi AM, Torpdahl M, Villa L, Graziani C, Hopkins K (2010). Evidence for a second genomic island conferring multidrug resistance in a clonal group of strains of *Salmonella enterica* serovar Typhimurium and its monophasic variant circulating in Italy, Denmark, and the United Kingdom. J. Clin. Microbiol.

[b23] Malorny B, Paccassoni E, Fach P, Bunge C, Martin A, Helmuth R (2004). Diagnostic real-time PCR for detection of *Salmonella* in food. Appl. Environ. Microbiol.

[b24] Mossong J, Marques P, Ragimbeau C, Huberty-Krau P, Losch S, Meyer G (2007). Outbreaks of monophasic *Salmonella enterica* serovar 4,[5],12:i:- in Luxembourg, 2006. Euro. Surveill.

[b25] Soyer Y, Moreno Switt A, Davis MA, Maurer J, McDonough PL, Schoonmaker-Bopp DJ (2009). *Salmonella enterica* serotype 4,5,12:i:-, an emerging *Salmonella* serotype that represents multiple distinct clones. J. Clin. Microbiol.

[b26] Swaminathan B, Gerner-Smidt P, Barrett T (2006). Focus on *Salmonella*. Foodborne Pathog. Dis.

[b27] Switt AI, Soyer Y, Warnick LD, Wiedmann M (2009). Emergence, distribution, and molecular and phenotypic characteristics of *Salmonella enterica* serotype 4,5,12:i. Foodborne Pathog. Dis.

[b28] Tavechio AT, Ghilardi AC, Fernandes SA (2004). “Multiplex PCR” identification of the atypical and monophasic *Salmonella enterica* subsp. *enterica* serotype 1,4,[5],12:i:- in Sao Paulo State, Brazil: frequency and antibiotic resistance patterns. Rev. Inst. Med. Trop. Sao Paulo.

[b29] de la Torre E, Zapata D, Tello M, Mejia W, Frias N, Garcia Pena FJ (2003). Several *Salmonella enterica* subsp. *enterica* serotype 4,5,12:i:- phage types isolated from swine samples originate from serotype Typhimurium DT U302. J. Clin. Microbiol.

[b30] Trupschuch S, Laverde Gomez JA, Ediberidze I, Flieger A, Rabsch W (2010). Characterisation of multidrug-resistant *Salmonella* Typhimurium 4,[5],12:i:- DT193 strains carrying a novel genomic island adjacent to the *thrW* tRNA locus. Int. J. Med. Microbiol.

[b31] Yamamoto S, Kutsukake K (2006). FljA-mediated posttranscriptional control of phase 1 flagellin expression in flagellar phase variation of *Salmonella enterica* serovar Typhimurium. J. Bacteriol.

